# Dr. J. N. Farrar

**Published:** 1913-08-15

**Authors:** 


### OBITUARY.

### Dr. J. N. Farrar.

John Nutting Farrar, D. D. S., M. D., died at his residence 1271 Broadway, New York City, June 12th. Dr. Farrar was born at Pepperall, Mass., 1839. He received an academic education and studied dentistry in the Pennsylvania College of Dental Surgery, graduating in 1865. He later graduated from the Jefferson Medical College.

Through his writings, especially on Orthodontia, he occupied a prominent position in the literature and history of the dental profession. His work, "Irregularities of the Teeth and Their Correction," containing nearly 2,000 illustrations is the most pretentious work on this subject ever published. He contributed considerable to dental journalism. His methods in Orthodontia, though possessing novelty in some respects—and for which due credit should be accorded—are now to be viewed more as a link in the progress of the art between the primitive methods of the past of those of the more advanced of the present time.

Dr. Farrar was a man of versatility of talent. He was a skilled mechanician, and a student of geneology. He traced his own line of descent back to that of William the Conqueror and included in it the English branch of the family of Washington. He was a man of genial disposition and gracious manners. He was beloved by those close to him and respected by all who knew him. He was complimented a few years ago by a dinner at the Brevoort Hotel given for him by the dentists of New York and New Jersey, which was largely attended.

He was a type of those men further back in the past, such as Parmly, Dunning, Allen, Roberts, Atkinson, Hawes, Dwinelle, and still more recently, our beloved Perry—with whom he was college classmate, who in New York City by their labors and course of professional life have done so much in many ways to advance dentistry as an art and science and obtain for it the position and respect as a profession it receives at the present time.

Dr. Farrar married in 1875. His wife survives him. He had but one child, a son, who died in infancy. Dr. Farrar's death was sudden. Though not in good health for some time he was in his office as usual on the 11th, was taken with an attack of nausea that evening, during which a cerebral hemorrhage occurred.—G. E., in *Dental Scrap Book.*

